# Comparability of dental implant site ridge measurements using ultra-low-dose multidetector row computed tomography combined with filtered back-projection, adaptive statistical iterative reconstruction, and model-based iterative reconstruction

**DOI:** 10.1007/s11282-018-0350-z

**Published:** 2018-10-13

**Authors:** Asma’a Abdurrahman Al-Ekrish, Reema Al-Shawaf, Wafa Alfaleh, Romed Hörmann, Wolfgang Puelacher, Gerlig Widmann

**Affiliations:** 1 Division of Oral and Maxillofacial Radiology, Department of Oral Medicine and Diagnostic Sciences, College of Dentistry, King Saud University, PO Box 60169, Riyadh, 11545 Saudi Arabia; 2 Division of Clinical and Functional Anatomy, Medical University of Innsbruck, Müllerstrasse 59, 6020 Innsbruck, Austria; 3 Department of Craniomaxillofacial Surgery, Medical University of Innsbruck, Anichstraße 35, 6020 Innsbruck, Austria; 4 Department of Radiology, Medical University of Innsbruck, Anichstraße 35, 6020 Innsbruck, Austria

**Keywords:** Anatomy, cross-sectional, Image-guided surgery, Imaging, three-dimensional, Multidetector row computed tomography, Radiation dosage

## Abstract

**Objective:**

To assess the linear measurements of edentulous ridges recorded from multidetector row computed tomography (MDCT) images obtained by a previously untested ultra-low dose in combination with filtered back-projection (FBP), adaptive statistical iterative reconstruction (ASIR), and model-based iterative reconstruction (MBIR).

**Methods:**

Three cadavers were imaged using a reference protocol with a standard dose and FBP (volume CT dose index (CTDIvol): 29.4 mGy) and two ultra-low-dose protocols, LD1 and LD2 (CTDIvol: 0.53 and 0.29 mGy). All test examinations were reconstructed with FBP, ASIR 50, ASIR 100, and MBIR. Linear measurements from the images of the edentulous ridges recorded from the test protocols were compared with those from the reference using a one-sample *t* test, Bland–Altman plots, and linear regression. Statistical significance was set at a *p* value of 0.05.

**Results:**

The one-sample *t* test demonstrated a statistically significant difference between the measurements from the reference protocol and all test protocols. The difference was not clinically significant for the following three test protocols: LD1/FBP, LD1/ASIR 50, and LD2/FBP. Bland–Altman plots with linear regression showed no systematic variation between the measurements obtained with the reference protocol and these three test protocols.

**Conclusions:**

The lowest-dose protocol to demonstrate comparable measurements with a standard MDCT dose was CTDIvol 0.29 mGy with FBP. These results must be considered with caution for areas of the jaws with thin cortication. The results in areas of thin cortication should be verified by studies with larger sample sizes at such areas and comparison with true gold standard measurements.

**Electronic supplementary material:**

The online version of this article (10.1007/s11282-018-0350-z) contains supplementary material, which is available to authorized users.

## Introduction

The widespread use of computed tomography (CT) in the analysis of prospective dental implant sites, although diagnostically beneficial, may contribute to the increasing collective dose of ionizing radiation to populations [1]. Therefore, dose optimization of multidetector row CT (MDCT) imaging in dental implantology is needed [2].

Although cone beam CT is advocated for replacement of MDCT in implant diagnostics because of the reportedly lower radiation dose and cost, MDCT is still the only option available in some settings. Radiation doses imparted by cone beam CT and MDCT are affected by numerous factors but may be comparable in some situations, especially if ultra-low MDCT doses are utilized [3–5].

One method that has been advocated because of its allowance of dose reductions in MDCT is the use of iterative reconstruction techniques (IRTs) to replace the filtered back-projection (FBP) technique in reconstruction of MDCT images. Two examples of IRTs are adaptive statistical iterative reconstruction (ASIR) and model-based iterative reconstruction (MBIR). Compared with FBP, the use of ASIR and MBIR have been shown to produce images with reduced noise at ultra-low doses [6]; however, they may also lead to over-smoothening of the images, which may adversely affect identification of anatomical interfaces in the images [7, 8]. Use of ASIR and MBIR, as well as FBP, with ultra-low-dose MDCT at a volume CT dose index (CTDIvol) of 0.53 mGy has been demonstrated to produce linear measurements of dental implant sites comparable with those produced with a standard dose (CTDIvol of 36.71 mGy); however, ASIR and MBIR did not provide an advantage over FBP [9].

Notably, most of the samples in the previous study were mandibular sites, which have thicker cortical boundaries than maxillary sites. It is therefore conceivable that identification of ridge boundaries and recording of ridge dimensions at maxillary sites may be more adversely affected at ultra-low MDCT doses and may thus benefit from reduced noise levels produced by ASIR and MBIR. Furthermore, the lowest dose limit for production of acceptable linear measurements and which reconstruction technique would support the lowest dose reductions remain unknown.

Investigation of more aggressive dose reductions using such techniques has the potential to further reduce the radiation doses imparted by MDCT imaging of dental implant sites. However, because the effect of dose on image quality is directly influenced by the reconstruction technique used, investigations of dose reductions must also take the reconstruction technique into consideration. Therefore, this study was performed to assess the comparability of linear measurements of edentulous ridges recorded from MDCT images obtained by a previously untested ultra-low dose in combination with FBP, ASIR, and MBIR compared with a reference MDCT protocol. Testing the various combinations of doses with reconstruction techniques will help to determine which reconstruction technique allows use of the lowest dose.

## Materials and methods

This study was performed using three cadaveric heads. Institutional review board approval was not required because the bodies used in the study were donated by people who had given their informed consent for their use for scientific and educational purposes prior to death and the study fulfilled all requirements necessary for studies on human cadavers according to the regulations of the Division of Clinical and Functional Anatomy, Medical University of Innsbruck [10, 11]. The cadavers had been preserved using an arterial injection of a formaldehyde–phenol/alcohol–glycerin solution and immersion in phenolic acid in water for 1–3 months [12]. Two of the cadavers were completely edentulous, and one was partially dentulous in the mandibular anterior/premolar regions.

### Imaging protocols

Each of the cadavers was imaged using a 64-row CT scanner (Discovery CT750 HD; GE Healthcare, Vienna, Austria) using a reference protocol and two ultra-low-dose test protocols, LD1 and LD2. The position of each cadaver within the gantry of the MDCT device was not changed between examinations. The exposure parameters, reconstruction technique, and imparted dose of each examination are detailed in Table 1. The effective doses of the protocols were also calculated from the dose–length product based on a *k* factor of 0.0021 for adult head examinations [13]. Nine MDCT datasets (1 reference and 8 test datasets) were obtained for each cadaver, for a total of 27 datasets. All datasets were obtained with a bone convolution kernel except for the MBIR datasets, which were obtained with a standard convolution kernel because a bone kernel is not available with MBIR. The datasets were uploaded in Digital Imaging and Communication in Medicine format to an online storage site, Google Drive; they were then downloaded and imported into a three-dimensional image reformatting software program (OnDemand Software, version 1.0; Cybermed Inc., Seoul, South Korea) for reformatting of the sample sites.

**Table 1:** List of exposure parameters and reconstruction techniques used in the reference and ultra-low-dose test protocols

Exposure protocol	Reconstruction technique	Matrix	In-plane voxel size (mm)	kV	mA	Rotation time (s)	Pitch	CTDIvol (mGy)	Effective dose (µSv)^a^	
									Scan length of 5 cm	Scan length of 10 cm
Reference	FBP	512 × 512	0.391	120	100	1.0	0.53	29.4	308.7	617.4
LD1	FBP	512 × 512	0.391	80	10	0.4	0.53	0.53	5.6	11.1
	ASIR 50									
	ASIR 100									
	MBIR	1024 × 1024	0.195							
LD2	FBP	512 × 512	0.391				0.97	0.29	3.0	6.1
	ASIR 50									
	ASIR 100									
	MBIR	1024 × 1024	0.195							

*CTDIvol* volume CT dose index, *LD* low-dose protocol, *FBP* filtered back-projection, *ASIR* adaptive statistical iterative reconstruction, *MBIR* model-based iterative reconstruction

^a^Calculated from the dose–length product based upon a *k* factor of 0.0021

### Image processing

The image processing and analysis method used was similar to that described in a previous study [9]. Processing and reformatting of all sample images were performed by one maxillofacial radiologist (A.A.) with 12 years of experience in MDCT image reformatting. The entire lengths of the maxillary and mandibular edentulous areas were used to obtain the cross-sectional sample images. The exclusion criteria for the sample sites were areas containing foreign objects or artificial defects and areas with buccolingual or occlusoapical dimensions of < 2 mm.

Each sample image was reformatted individually using the three-dimensional module of the OnDemand software. Because the position of each cadaver was fixed during the MDCT examinations, the default position and angulation of the sectional planes were identical in all datasets of each cadaver. Thus, the measured shift and angulation of the image planes allowed the production of sample sites with a standardized location and orientation between different examination protocols. The reformatting protocol for each cadaver is available upon request. The mA and kVp values were turned off, and each sample site was saved by bookmarking in the OnDemand software on the hard drive of the computer. Table 2 shows the images of one sample site obtained by the reference and test protocols.

**Table 2:**
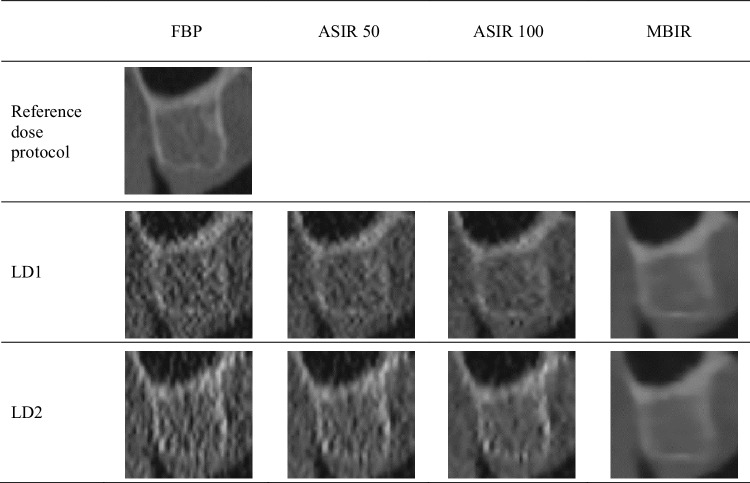
Images of a sample site obtained with the various combinations of exposure protocols and reconstructions techniques

*LD* low-dose protocol, *FBP* filtered back-projection, *ASIR* adaptive statistical iterative reconstruction, *MBIR* model-based iterative reconstruction

### Image analysis

Two oral and maxillofacial radiologists (R.S. and W.A.) with 8 and 14 years of experience, respectively, in interpretation of CT images recorded the test measurements. The examiners were blinded to the exposure protocols and reconstruction techniques and were calibrated in identification of the outer boundaries of the edentulous bone on cross-sectional images. The examiners viewed the images at a set window width/window level of 3000/650, but they were permitted to adjust the magnification level for clarity.

**Fig. 1:**
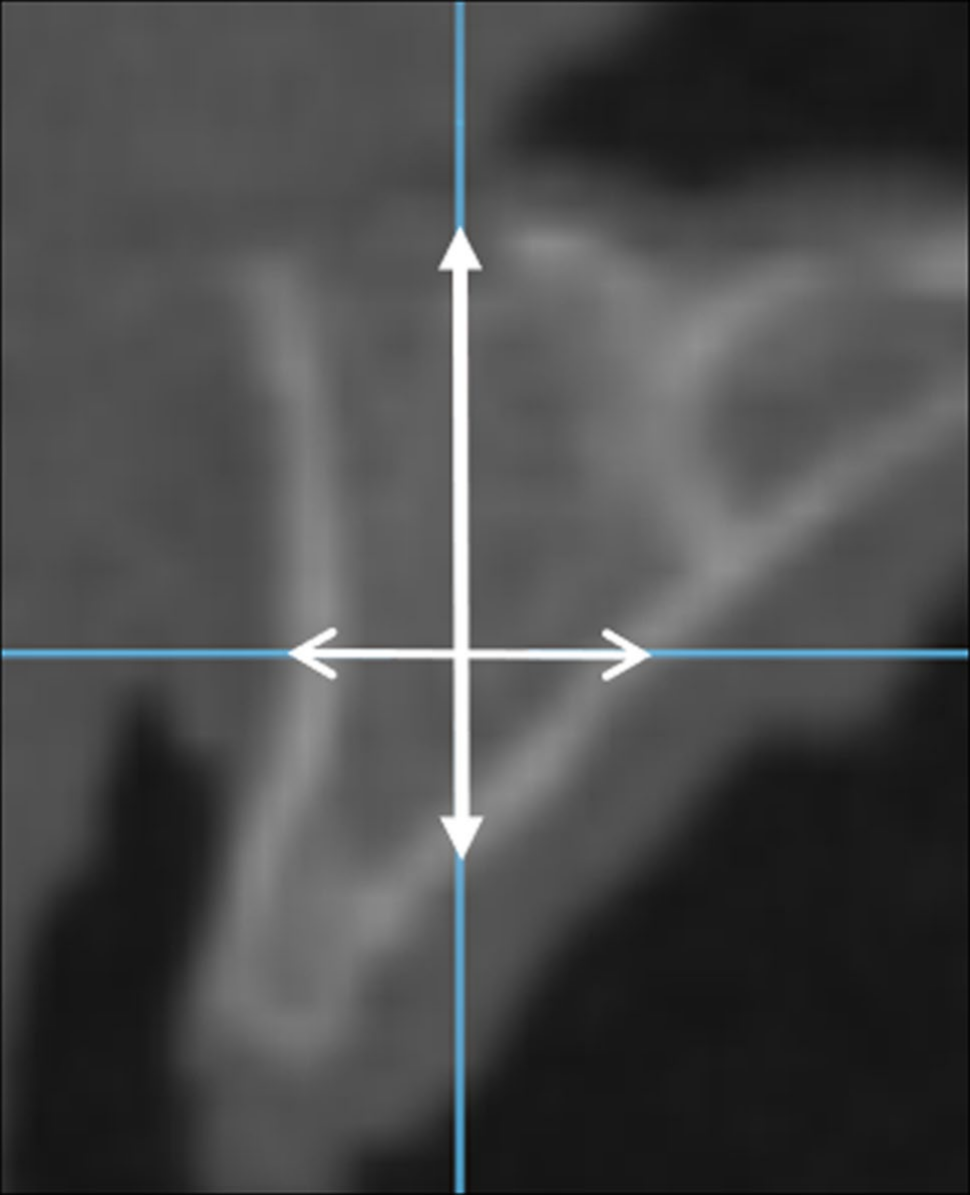
Sample cross-sectional image from the anterior maxilla (acquired with the reference protocol) demonstrating the location and direction of the height and width measurements. The height measurement was recorded along the line representing the parasagittal plane (marked by closed arrows) extending from the floor of the nasal cavity to the outer surface of the cortical plate. The width measurement was recorded along the line representing the axial plane (marked by open arrows) extending between the outer surfaces of the facial and lingual cortical plates

The height and width measurements of the ridge were recorded on the sample images along the lines representing the parasagittal and axial planes, respectively. The limits of the measurements were the outer cortical boundaries of the ridge. The apical cortical boundary at the anterior maxillary sites was the floor of the nasal cavity, and that at the posterior maxillary sites was the floor of the maxillary sinus (Fig. 1). The first examiner (R.S.) recorded all measurements from all of the samples once. Next, using a random number generator (http://stattrek.com/statistics/random-number-generator.aspx), 50 measurements were selected for reliability testing. These measurements were then repeated by the two examiners independently of each other.

### Statistical evaluation

The two measurements recorded by the first examiner were used to calculate intra-examiner reliability. For calculation of the inter-examiner reliability, the mean of the two measurements recorded by the first examiner was compared with the corresponding measurements recorded by the second examiner. Intra-examiner and inter-examiner reliability were analyzed by Cronbach’s alpha and inter-item correlation.

Agreement between the measurements recorded from the reference protocol and each of the test protocols was tested by subtracting the test measurements from the reference measurements and analyzing the difference with a one-sample *t* test (test value: zero). For each test protocol, if the one-sample *t* test revealed the absolute mean difference to be < 0.3 mm with a 95% confidence interval within ± 0.5 mm, then it was considered that no clinically significant difference was present; in such cases, a Bland–Altman plot was constructed and linear regression was used to test for proportional bias [14]. Statistical analysis was performed using the Statistical Package for the Social Sciences (SPSS) version 24 (IBM Corp., Armonk, NY, USA). Statistical significance was set at a *p* value of 0.05.

## Results

Thirty sample images were obtained from the three cadavers. The site distribution of the samples is shown in Table 3. Height and width measurements were recorded from each sample for a total of 60 measurements from each imaging protocol. Nine imaging protocols were used for each sample for a total of 540 measurements.

**Table 3:** Distribution of the sample sites

Cadaver	Maxillary sites			Mandibular sites			Total
	Incisor	Canine/premolar	Molar	Incisor	Canine/premolar	Molar	
1	1	2	2	2	–	4	11
2	1	2	2	1	–	4	10
3	1	2	2		1	3	9
Total	3	6	6	3	1	11	30

The intra-examiner and inter-examiner reliability of the measurements were found to be very high, with Cronbach’s alpha and intraclass correlation coefficients of 0.997 for the intra-examiner reliability and 0.998 for the inter-examiner reliability (*p* < 0.001).

The one-sample *t* test revealed a statistically significant difference in measurements between the reference protocol and all the test protocols (Table 4). However, the difference was not clinically significant for the following three test protocols: LD1/FBP, LD1/ASIR 50, and LD2/FBP. Bland–Altman plots (Electronic Supplementary Fig. 1) with linear regression (Table 4) showed no systematic variation between the measurements obtained with the reference protocol and these three test protocols.

**Table 4:** Differences between linear measurements obtained by the reference protocol (reference dose/filtered back-projection) and the various test protocols

	Mean difference (mm)*	Standard deviation of difference (mm)	95% confidence interval limits (mm)		Significance (linear regression)
			Lower limit	Upper limit	
LD1/FBP	− 0.2083	0.61128	− 0.3662	− 0.0504	0.605
LD1/ASIR 50	− 0.2817	0.66498	− 0.4534	− 0.1099	0.394
LD1/ASIR 100	− 0.3467	0.61959	− 0.5067	− 0.1866	–
LD1/MBIR	− 0.4667	1.01508	− 0.7289	− 0.2044	–
LD2/FBP	− 0.2500	0.76878	− 0.4486	− 0.0514	0.584
LD2/ASIR 50	− 0.6367	1.47269	− 1.0171	− 0.2562	–
LD2/ASIR 100	− 0.4050	0.55736	− 0.5490	− 0.2610	–
LD2/MBIR	− 0.4583	1.05899	− 0.7319	− 0.1848	–

– Bland–Altman plot and linear regression were not performed because the one-sample *t* test demonstrated a mean difference greater than ± 0.3 mm with 95% confidence limits greater than ± 0.5 mm

*LD* low-dose protocol, *FBP* filtered back-projection, *ASIR* adaptive statistical iterative reconstruction, *MBIR* model-based iterative reconstruction

*All mean differences were statistically significant (*p* < 0.05)

## Discussion

The present study was performed to test the accuracy of linear measurements of edentulous ridges recorded from previously untested ultra-low-dose MDCT images compared with those from a reference dose protocol. The effective doses achieved in the present study are lower than those reported for many cone beam CT devices, even for some small-field-of-view examinations [4]. The results indicate that the measurements recorded using the lowest dose tested, CTDIvol of 0.29, and FBP were comparable with those from a reference dose protocol. The IRTs of ASIR and MBIR did not allow similarly accurate measurements. As such, the present study demonstrates previously unrealized potentials of ultra-low reduction in doses and FBP in implant site imaging.

The images of some sample sites, especially in the maxillary posterior regions, had very thin cortical boundaries and thus subjectively appeared to be more adversely affected by the amount of noise seen with the LD2/FBP protocol. However, the boundaries of the ridge were still identifiable in the images acquired by LD2/FBP as evidenced by the objective measurements presented in Table 4. This finding, namely that clinically useful information may be obtained with images that are not subjectively pleasing to the eye, was addressed by the National Council on Radiation Protection and Measurements when they modified the ALARA concept of imaging (as low as reasonably achievable) to the ALADA concept (as low as diagnostically achievable) [2]. The modification is meant to emphasize that regardless of whether the images are subjectively pleasing to the eye, the lowest doses should be used provided that the diagnostic information is obtainable. The European Union Council Directive 2013/59/EURATOM also addressed this issue by mandating that for all imaging procedures, the dose of ionizing radiation should be kept as low as reasonably achievable, consistent with obtaining the required medical information [15].

The improved performance of FBP over ASIR and MBIR in the present study occurred despite smaller voxel sizes being used with MBIR. The poorer performance of ASIR and MBIR compared with FBP may be due to their over-smoothening effect, which may increase the area of blurriness at the margins of the bone [7, 8]. For the same reason, previous studies have shown that ASIR and MBIR did not improve spatial resolution or the detection of fractures [8, 16] or identification of the roof of the inferior alveolar canal [17].

In the present study, the clinical significance of the absolute measurement differences was designated as > 0.3 mm with a 95% confidence interval beyond ± 0.5 mm for several reasons. First, this range of error is smaller than the size of the image voxel. Second, the measurement error of the reference protocol images when compared with the gold standard of measurements recorded directly from the bone is expected to be larger than this value; previous studies that compared ridge dimensions from MDCT images acquired with the standard protocol versus the dimensions recorded directly from bone showed that the smallest mean error was 0.36 mm with a standard deviation of ± 0.24 mm [18–21]. Third, implant diameters may include 0.3-mm increments, and during interpretation of images, operators commonly round off linear measurements to the nearest 0.5 mm.

The test protocols in the present study utilized the lowest mA and kV settings possible with the device used. Thus, to further reduce the radiation dose, the rotation time was reduced to less than half of that used for the reference protocol. The combined effect of reduced mA and rotation time is a reduction in the amount of X-ray photons, but an increase in the quantum noise in the resultant images. However, a previous study demonstrated accurate ridge dimensions using the same mA and kV and rotation time settings as LD1 [9]. Therefore, to further lower the dose, the present study used a higher pitch value in LD2 than that used in the previous study. The pitch value used in the present study did not exceed the maximum acceptable pitch values for 64-row MDCT scanners, namely 0.9844, beyond which interpolation of data occurs [22]. Although some researchers reported that the use of a pitch factor of 2 did not reduce the subjective quality of MDCT images of implant sites when combined with standard exposure parameters [23], other investigators reported that an increasing pitch factor was negatively associated with the number of successful objective linear measurements [24]. However, considering the high degree of comparability between the reference and lowest-dose protocol in the present study, investigation of further dose optimization using further increases in pitch is recommended.

The results of the present study are in agreement with a previous study that revealed comparable measurements between a reference dose protocol and an ultra-low-dose protocol similar to LD1 of the present study [9]. However, the previous study indicated that ASIR and MBIR produced measurements comparable to FBP, whereas the present study showed that FBP was more accurate at such very low doses. The reason for the differences may be that in the previous study, the sample sites were mostly in the mandibular molar region, which has a thick layer of dense cortical bone that is more clearly depicted in images and does not require image contrast as high as that needed for thin, low-density bone, which was found in other parts of the jaws in the present study. Thus, although ASIR and MBIR are beneficial in terms of noise reduction, FBP images should be used for bone measurements.

A limitation of the present study is that a site-specific analysis of the measurement differences was not conducted because of the small sample size in each specific area of the jaws. Although the overall study sample demonstrated no clinically significant difference with three of the test protocols, larger clinically significant differences might occur at particular sites with thin, low-density margins. As such, further studies with larger sample sizes at each area of the jaws and separate analysis of the measurements from each area are recommended. Additionally, the present study did not evaluate the influence of various sharpness levels of convolution kernels using ultra-low-dose images and IRTs. The use of various sharpness levels might produce different results than those obtained in the present study.

Another limitation of the present study is that the test measurements were not compared with a true gold standard of direct bone measurements. Using the reference protocol measurements as the standard must be considered with caution because such measurements have been shown to have statistically significant error [21]. Whether the measurement differences between the test and reference protocols in the present study would translate to increased or decreased error compared with bone measurements remains unknown. Therefore, further studies are recommended to investigate the absolute error of measurements compared with a true gold standard.

The lowest-dose protocol to demonstrate comparable measurements with a standard MDCT dose was found to be a CTDIvol of 0.29 mGy with FBP. The IRTs of ASIR and MBIR yielded larger measurement differences. These results must be considered with caution when evaluating areas of the jaws with thin cortication. The results in such areas should be verified by studies with larger sample sizes in such areas, and the findings should be compared with true gold standard measurements.

## Electronic supplementary material

Below is the link to the electronic supplementary material.

Supplementary material 1 (DOCX 57 KB)
