# Supplementary material for: Comparability of dental implant site ridge measurements using ultra-low-dose multidetector row computed tomography combined with filtered back-projection, adaptive statistical iterative reconstruction, and model-based iterative reconstruction
Source: Oral Radiol. 2018 Oct 13;35(3):280–6. doi: 10.1007/s11282-018-0350-z (PMC6685921; doi:10.1007/s11282-018-0350-z)

**Dose Optimization for Dental Implant Site Measurements using MDCT with FBP, ASIR, and MBIR**

1. Asma'a Al-Ekrish
2. Reema Shawaf
3. Wafa Al-Faleh
4. Romed Hoerman
5. Wolfgang Puelacher
6. Gerlig Widmann

**Electronic Supplementary Figure 1**

**Bland-Altman plots of the measurement differences between the reference protocol and the three test protocols for which the One-sample t-test did not demonstrate a clinically significant difference (LD: Low dose protocol). The horizontal lines indicate the mean of the overall differences and the 95 % limits of agreement (standard deviation x 1.96).**

**Reference- LD1/FBP**


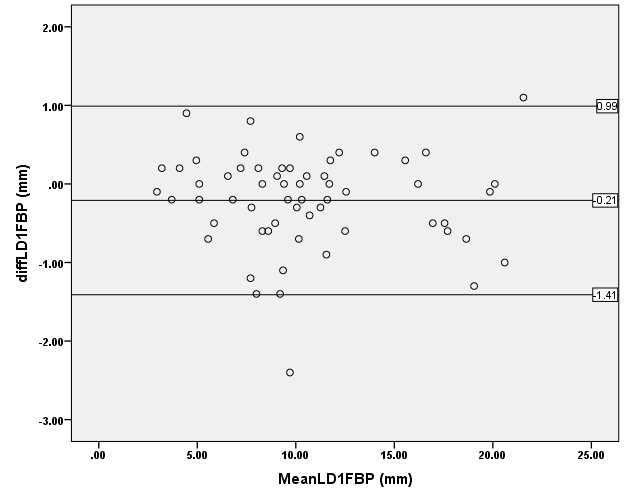


**Reference- LD1/ASIR 50**


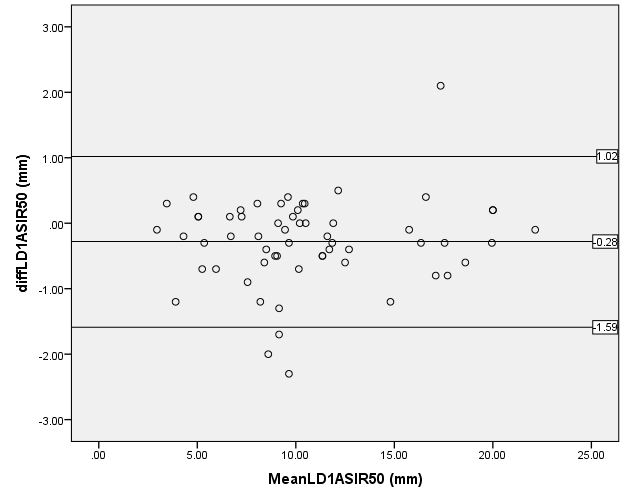


**Reference- LD2/FBP**


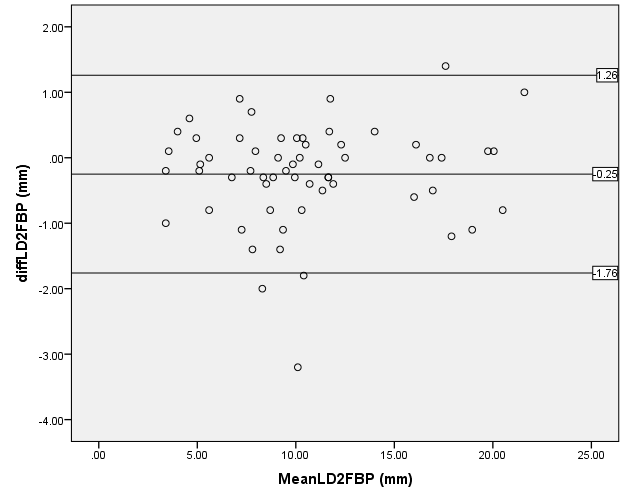

Supplement: Supplementary file 1 — Supplementary material 1 (DOCX 57 KB) [file 11282_2018_350_MOESM1_ESM.docx]
